# Contrast-enhanced transrectal ultrasound can reduce collection of unnecessary biopsies when diagnosing prostate cancer and is predictive of biochemical recurrence following a radical prostatectomy in patients with localized prostate cancer

**DOI:** 10.1186/s12894-020-00659-6

**Published:** 2020-07-16

**Authors:** Hong-wei Zhao, Jian Li, Jia-Zheng Cao, Juan Lin, Zhu Wang, Jian-yao Lv, Jin-huan Wei, Zhen-hua Chen, Hao-hua Yao, Yi-hui Pan, Zhen-li Gao, Jun-hang Luo, Wei Chen, Lei Shi, Yong Fang

**Affiliations:** 1 Department of Urology, Affiliated Yantai Yuhuangding Hospital, Qingdao University, Yantai, 264000 Shandong P. R. China; 2 Department of Urology, First Affiliated Hospital, Sun Yat-Sen University, No.58 ZhongShan 2nd Road, Guangzhou, 510080 Guangdong P. R. China; 3 State Key Laboratory of Oncology in South China, Department of Ultrasound, Sun Yat-Sen University Cancer Center, Guangzhou, 510060 Guangdong P. R. China; 4 Department of Urology, Jiangmen Hospital, Sun Yat-Sen University, Jiangmen, 529000 Guangdong P. R. China; 5 Department of Pediatrics, hird Affiliated Hospital of Sun Yat-sen University, Guangzhou, 510630 Guangdong P. R. China; 6 Department of Ultrasound, First Affiliated Hospital, Sun Yat-Sen University, Guangzhou, 510080 Guangdong P. R. China

**Keywords:** Contrast-enhanced, Transrectal ultrasound, Prostate cancer, Unnecessary prostate biopsy, Biochemical recurrence

## Abstract

**Background:**

To investigate the value of using contrast-enhanced transrectal ultrasound (CETRUS) to reduce unnecessary collection of biopsies during prostate cancer diagnosis and its utility in predicting biochemical recurrence in patients with localized prostate cancer.

**Methods:**

This was a prospective study of suspected prostate cancer patients who were evaluated with CETRUS followed by a prostate biopsy. Prostate blood flow via CETRUS was graded using a 5-point scale. The relationship between CETRUS score and biopsy outcome was then analyzed for all patients; univariate and multi-variate analyses were used to determine the probable prognostic factors for biochemical recurrence in patients with localized prostate cancer that underwent a radical prostatectomy.

**Results:**

A total of 347 patients were enrolled in the study. Prostate cancer was found in 164 patients. A significant positive correlation (r = 0.69, *p* < 0.001) was found between CETRUS scores and prostate cancer incidence. Using CETRUS scores ≥2 as the threshold for when to biopsy could have safely reduced the number of biopsies taken overall by 12.1% (42/347) and spared 23.0% (42/183) of patients from undergoing an unnecessary biopsy. 77 patients with localized prostate cancer underwent a radical prostatectomy. The median follow-up time was 30 months (range: 8–56 months) and 17 of these 77 patients exhibited biochemical recurrence during the follow-up period. 3-year biochemical recurrence-free survival rates were 86% for patients with low CETRUS scores (≤ 3) and 59% for patients with high scores (> 3; *p* = 0.015). Multivariate Cox regression analysis indicated that CETRUS score was an independent predictor of biochemical recurrence (HR: 7.02; 95% CI: 2.00–24.69; *p* = 0.002).

**Conclusions:**

CETRUS scores may be a useful tool for reducing the collection unnecessary biopsy samples during prostate cancer diagnosis and are predictive of biochemical recurrence in patients with localized prostate cancer following a radical prostatectomy.

## Background

Prostate cancer is the most common solid neoplasm and the second leading cause of cancer death in men in the US [1]. Both incidence and mortality are increasing, with an estimated 60,300 new cases diagnosed and 26,600 prostate cancer-related deaths in China in 2015 [2]. Approximately one million prostate biopsies are conducted per year in the US. However, prostate-specific antigen (PSA) testing, the most widely used screening test for prostate cancer, leads to 750,000 unnecessary biopsies—and accompanying pain, inconvenience, financial burden, and risk of infection [3]. Therefore, one way to reduce the use of PSA testing and shift the ratio of benefit to harm in favor of patient benefit would be to improve alternate methods with predictive value and thereby reduce unnecessary biopsy collection [4].

About 15–40% of patients with localized prostate cancer will suffer recurrence following a radical prostatectomy [5]. Traditional clinicopathologic risk factors, such as serum PSA levels, pathological grading (Gleason score), and TNM staging are used to predict the probability of recurrence in prostate cancer patients, but these tools are not accurate for every patient [6]. Therefore, new tools are needed to complement the prognostic value of traditional risk factors. Such tools may help guide individual therapeutic management, improve patient counseling, and help optimize the design of clinical trials.

Previous studies have shown that contrast-enhanced transrectal ultrasound (CETRUS)-based blood flow grading is a reliable tool for predicting pathological outcomes of prostate-related diseases [7–11]. The diagnostic accuracy of CETRUS has continually improved with the development of new ultrasound contrast agents and equipment [12]. Although CETRUS is a very promising imaging tool, it is still not widely used to help diagnose or evaluate prostate cancer. In this study, we investigated whether CETRUS can reduce unnecessary biopsy collection during prostate cancer diagnosis. We also investigated whether CETRUS can predict biochemical recurrence following a radical prostatectomy in patients with localized prostate cancer.

## Methods

### Patient selection

Patients referred for a prostate biopsy based on elevated total serum PSA levels (> 4 ng/mL) or abnormal digital rectal examination (DRE) between Feb 2014 and Sep 2018 at our collaborator institutions (Yantai Yuhuangding Hospital, First Affiliated Hospital of Sun Yat-sen University and Cancer Center of Sun Yat-sen University) were included in the study. The exclusion criteria were as follows: (1) age > 80 years; (2) refusal of CETRUS screening; (3) patient had received a previous prostate biopsy; (4) patient had a severe cardiopulmonary disease. The study was approved by the ethics committee of the institutions involved. Written informed consent to participate in the study and for the publication of their individual data was obtained from each participant enrolled in the study.

### Procedures

CETRUS was performed for each patient before prostate biopsy collection. The ultrasound equipment used for CETRUS was an IU 22 system (Philips, Holland), and the contrast agent used was SonoVue (Bracco, Milan, Italy). Conventional and contrast-enhanced transrectal ultrasound examinations were performed by sonographers that had more than 5 years of experience in conducting contrast-enhanced ultrasounds; these sonographers were not involved in the analysis of ultrasound images. All patients were examined in the left lateral position. A bolus of SonoVue (2.4 mL) was injected intravenously, followed by a 5 mL saline flush to ensure no residual contrast agent remained in the intravenous catheter. A series of axial sweeps through the gland were obtained from base to apex, with each sweep having a duration of 20–30 s [13]. This process was continuously observed for at least 3 min. Repeated injections (2.4 mL) were performed in 16 patients when initial results were unsatisfactory. All examinations at a given center were performed by the same sonographers. All CETRUS images were simultaneously examined by two sonographers. All sonographers involved were blinded to the patients’ clinical presentations and other imaging results.

Diagnostic confidence was scored on a five-point scale based on blood flow, according to previously published reports: [8, 13] score 1, definitely benign, minimal enhancement (capsular and periurethral flow only); score 2, probably benign, mild enhancement (symmetric radial flow from capsular branches); score 3, indeterminate, mildly increased enhancement (asymmetric/increased flow in the prostate); score 4, probably malignant, moderately increased enhancement (asymmetric/increased flow in the prostate); score 5, definitely malignant, substantially increased enhancement (asymmetric/increased flow in the prostate) (Additional Fig. 1).

After CETRUS examination, a systematic 12-core prostate biopsy was performed. The systematic biopsy strategy consisted of collection of 6 standard cores and an additional 3 cores positioned more laterally on each side. Each biopsy sample was reviewed by a urological pathologist and reported individually. Tumors were classified according to the 2002 TNM staging system and graded according to the Gleason grading system. High-grade cancer was defined as a Gleason score 8 or higher.

### Surgical management and follow-up

Patients with clinical stage T1 and T2 prostate cancer underwent a laparoscopic radical prostatectomy. Patients with pathologically diagnosed localized prostate cancer (T1/T2N0M0) were enrolled and followed up prospectively. Data collected from each patient included age at diagnosis, CETRUS score, tumor stage, Gleason score, body-mass index (BMI), Eastern Cooperative Oncology Group performance status (ECOG PS), and biochemical recurrence-free survival time. Postoperatively, patients were examined to obtain serum PSA measurements every 3 months for the first year following surgery and semi-annually from the second through the fifth years. Biochemical recurrence was defined as a PSA level ≥ 0.2 ng/mL and rising, and recurrence date was assigned to the first instance of a PSA value ≥0.2 ng/mL [6]. No patient received adjuvant therapy prior to development of biochemical recurrence. Biochemical recurrence-free survival was calculated from the date of surgery to the date of biochemical recurrence and was concluded at the date of death from other causes or the date of the last follow-up visit.

### Statistical analysis

The correlation between CETRUS blood flow grades and biopsy outcomes were analyzed using a Spearman correlation test. Two groups were compared using the chi-square test for categorical variables. Receiver operating characteristic (ROC) curves and area under the curve (AUC) determinations were employed to assess the diagnostic accuracy of CETRUS. Survival curves were estimated using the Kaplan-Meier method. The univariate Cox proportional regression hazard model was used to analyze the correlation between variables and clinical outcomes. Multivariate survival analysis was performed on all parameters that were significant by univariate analysis using the Cox regression model. The predictive accuracy of prognostic factors was determined using time-dependent ROC analysis, and the AUC at 3 years post-surgery were used to measure predictive accuracy. R software version 2.7.1 (R Foundation for Statistical Computing, Vienna, Austria) was used for time-dependent ROC analysis. SPSS software (SPSS Standard version 16.0; SPSS Inc., Chicago, IL, USA) was used for all other calculations. Statistical significance was indicated by *p* < 0.05.

## Results

### Patient characteristics

**Fig. 1:**
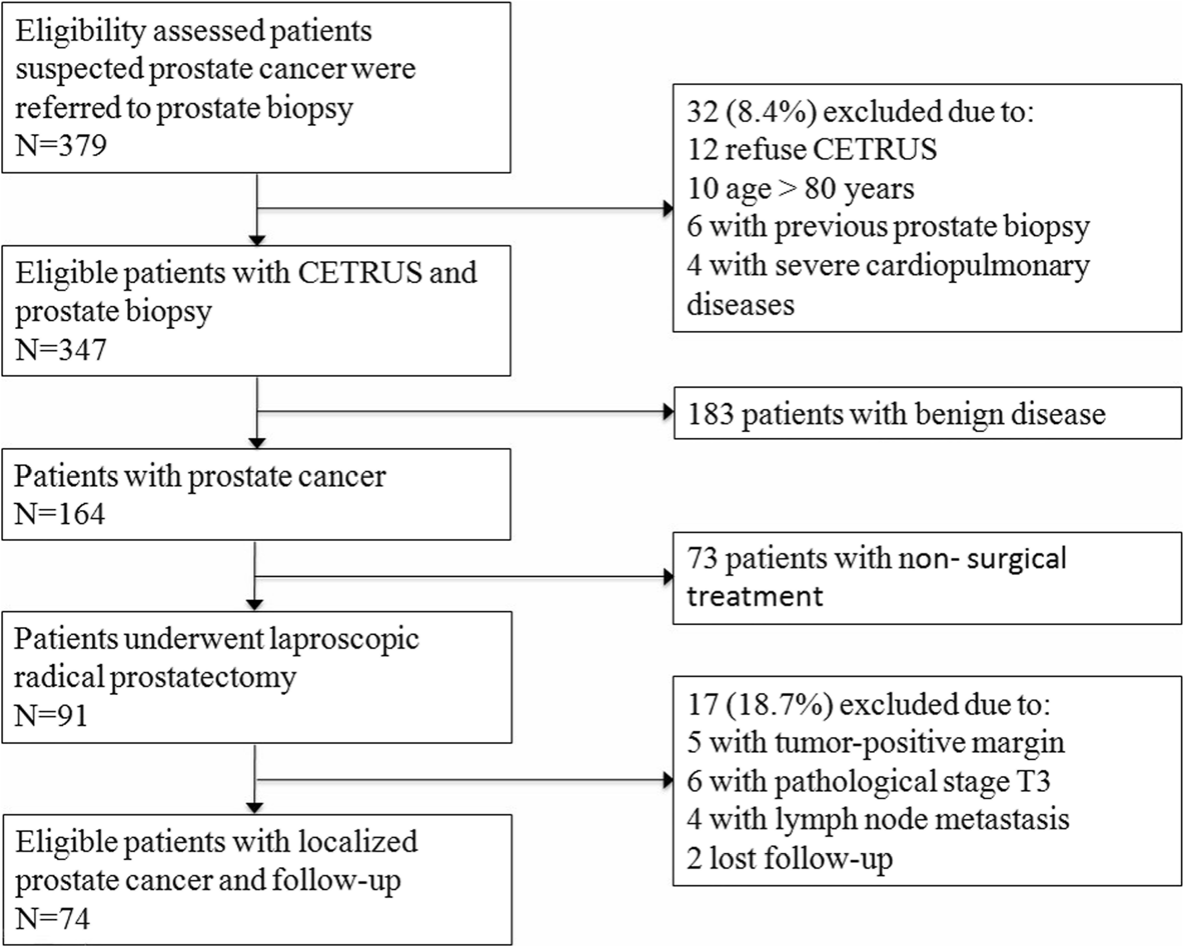
Flowchart for selection of patients for CETRUS examination and follow up

A total of 379 consecutive patients suspected of prostate cancer were referred for a prostate biopsy. Thirty-two patients were excluded. 347 patients were enrolled into the study and evaluated with CETRUS followed by collection of prostate biopsy samples. No adverse events related to the contrast agent were observed in any of the 347 patients. The mean patient age was 68.5 years (range: 53–79), mean prostate volume was 52.8 mL^3^ (range: 18–136), and median the PSA level was 8.9 ng/mL (range: 1.9–382). The number of men with a PSA level < 4, 4–10, 10–20 and > 20 were 28, 160, 116, and 43, respectively. Prostate cancer was found in a total of 164 of 347 (47.3%) patients. The proportion of patients with Gleason scores ≤6, =7 and ≥ 8 were 22.6% (37), 43.3% (76), and 31.1% (51), respectively. Among the 164 patients, 91 patients underwent a laparoscopic radical prostatectomy. Seventeen patients were excluded. Finally, 74 patients with localized prostate cancer were enrolled and followed up as shown in Fig. 1. Of the patients treated with a radical prostatectomy, the proportion of patients with Gleason scores ≤6, =7 and ≥ 8 were 24.7% (19), 41.6% (32), and 33.7% (26), respectively.

### CETRUS is a useful tool for reducing the performance of unnecessary biopsies

**Fig. 2:**
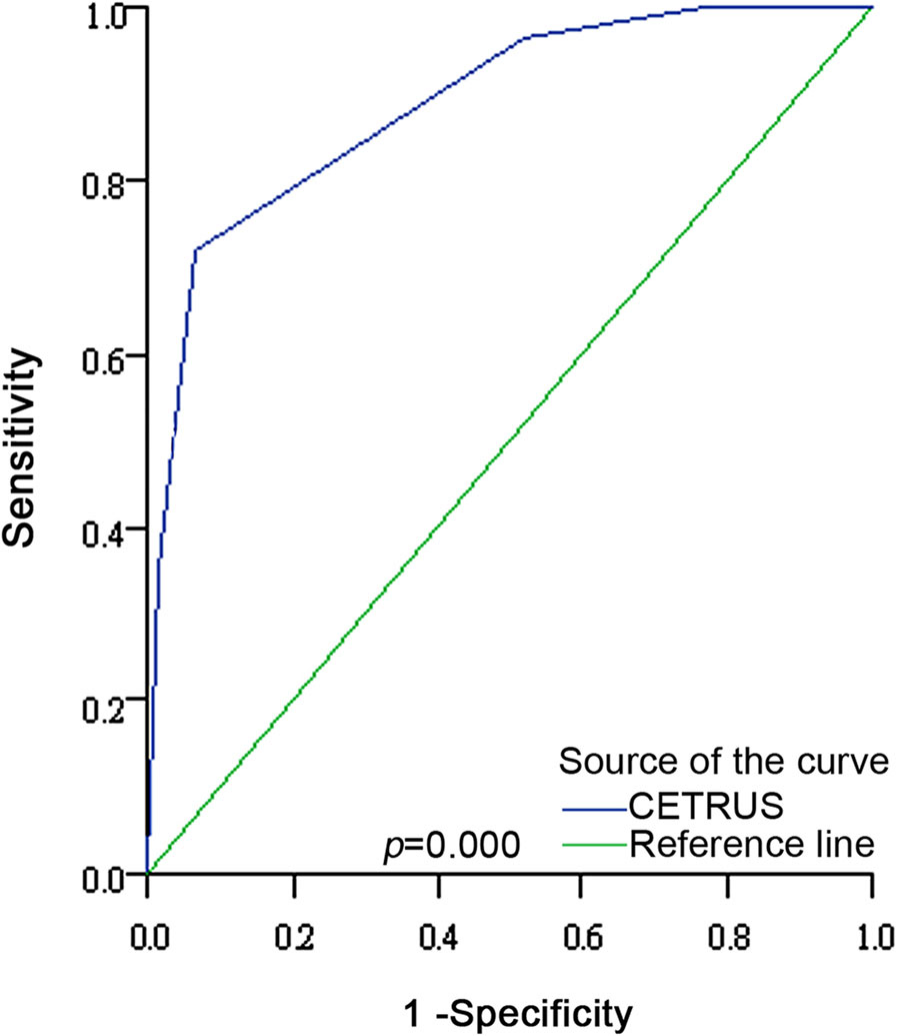
ROC curve using CETRUS scores to differentiate prostate cancer from benign disease. The AUC for the CETRUS scores was 0.89 (95% CI: 0.85–0.92, *p* < 0.0001)

The percentage of prostate cancer patients in CETRUS score groups 1–5 were 0% (0/42), 11.5% (6/52), 32.5% (40/123), 87.1% (61/70), and 95.0% (57/60), respectively. A significant positive correlation (r = 0.69, *p* < 0.001) was found between CETRUS scores and prostate cancer incidence. When cut off at 4 (≤ 3 as BPH and ≥ 4 as prostate cancer), the CETRUS score had an accuracy of 83.3% (289/347) in correctly diagnosing prostate cancer, with a sensitivity and specificity of 90.8% (118/130) and 78.8% (171/217), respectively. ROC analysis showed the AUC of the CETRUS score was 0.89 (95% CI: 0.85–0.92), indicating that patient CETRUS scores can be used to differentiate prostate cancer from benign prostatic hyperplasia (BPH) (Additional Table 1, Fig. 2). Moreover, when cut off at 2 (1 = BPH and ≥ 2 as prostate cancer), CETRUS scores demonstrated a specificity of 100% for differentiating prostate cancer from BPH, indicating that application of CETRUS using a cut-off score of 2 might be an effective tool for reducing the performance of unnecessary prostate biopsies. In this study, performing biopsy for cases with a CETRUS score ≥ 2 could have reduced the number of biopsies by 12.1% (42/347) without missing any cancer diagnoses and spared 23.0% (42/183) of men from undergoing an unnecessary biopsy (Table 1).

**Table 1:** Efficacy of CETRUS with a cut-off score of 2 in reducing unnecessary prostate biopsies

Biopsy strategy	No. of biopsies	No. of necessary biopsies^a^	No. of unnecessary biopsies^a^
Conventional standard	347	164	183
Combining CETRUS score ≥ 2^b^	305	164	141
Reduction in the no. of biopsies	42	0	42
Proportion of the reduced biopsies	12.1% (42/347)^c^	0% (0/164) ^c^	23.0% (42/183)

^a^ Necessary biopsies: performed for malignant disease as confirmed by pathologic evaluation; Unnecessary biopsies: performed for benign disease as confirmed by pathologic evaluation

^b^ Conventional standard of biopsy combining CETRUS blood flow scale score ≥ 2

^c^ Proportion of reduced biopsies without missing cancer

### CETRUS can predict biochemical recurrence following a radical prostatectomy in patients with localized prostate cancer

The clinicopathologic characteristics of the 77 patients diagnosed with localized prostate cancer treated with a radical prostatectomy are summarized in Table 2. CETRUS score results were categorized in low score (≤ 3) and high score (> 3) groups. No significant correlation was found between CETRUS score and patient age, tumor stage, Gleason score, PSA level, BMI, or ECOG PS (*p* > 0.05, Table 2).

**Table 2:** Association of CETRUS scores with the clinicopathologic characteristics of 77 patients with localized prostate cancer who underwent radical prostatectomy

Variables	No.	Low score (score ≤ 3)	High score (score > 3)	*p*
Age (years)				0.834
≤ 65	43	20 (46.5%)	23 (53.5%)	
> 65	34	15 (44.1%)	19 (55.9%)	
Tumor stage				0.756
T1N0M0	25	12 (48.0%)	13 (52.0%)	
T2N0M0	52	23 (44.2%)	29 (55.8%)	
Gleason score				0.291
≤ 7	51	21 (41.2%)	30 (58.8%)	
> 7	26	14 (53.8%)	12 (46.2%)	
PSA (ng/mL)				0.611
< 15	46	22 (47.8%)	24 (52.2%)	
≥ 15	31	13 (41.9%)	18 (58.1%)	
BMI (kg/m^2^)				0.802
< 25	43	19 (44.2%)	24 (55.8%)	
≥ 25	34	16 (47.1%)	18 (52.9%)	
ECOG PS				0.567
0	51	22 (43.1%)	29 (56.9%)	
≥ 1	26	13 (50.0%)	13 (50.0%)	
Total	77	35 (45.5%)	42 (54.5%)	

**Fig. 3:**
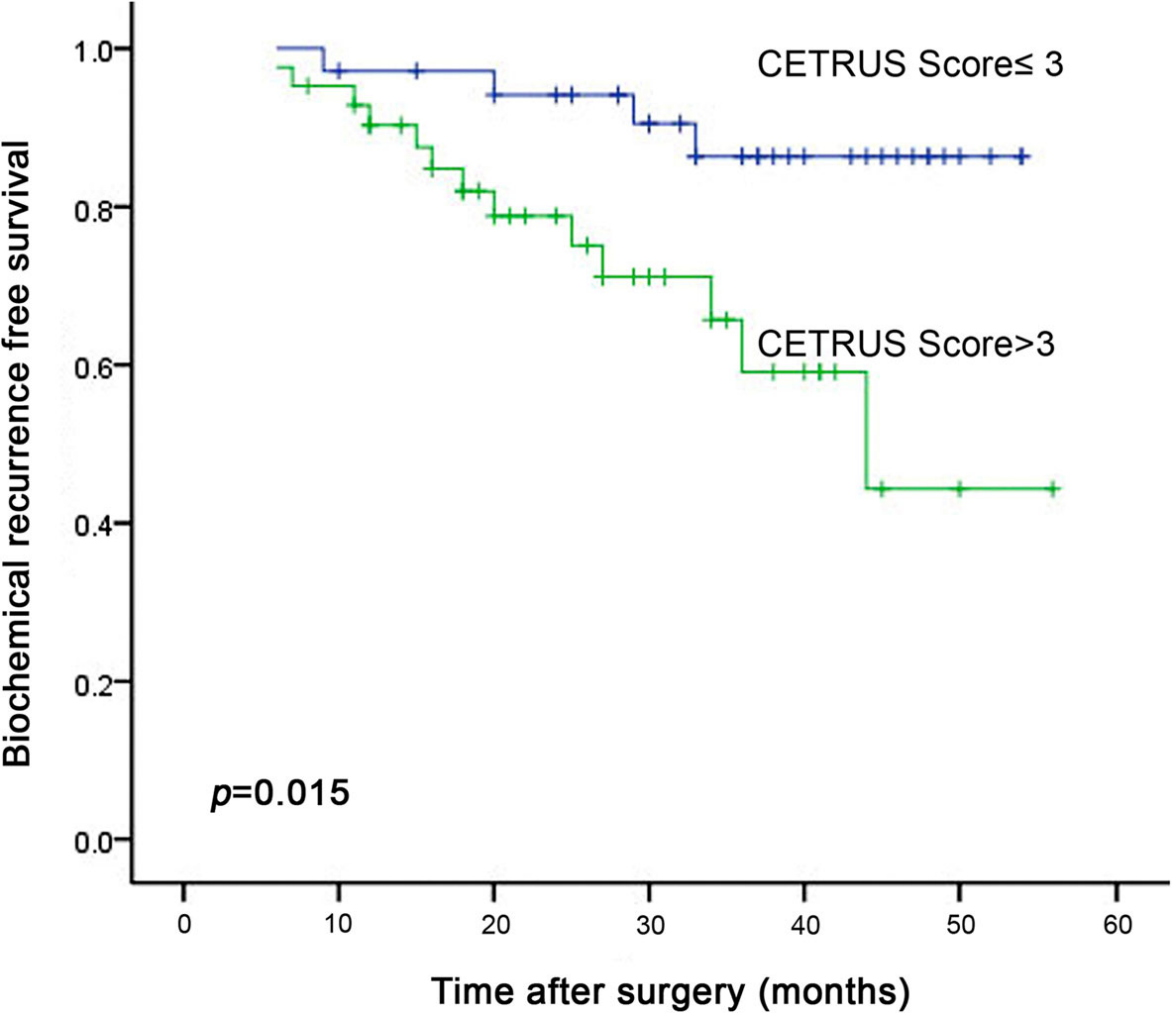
Kaplan-Meier biochemical recurrence-free survival analysis according to CETRUS scores for 77 patients with localized prostate cancer who underwent a radical prostatectomy (*p* = 0.015)

The median age of the 77 patients was 65.1 years (range: 49–74 years), and the median follow-up time after surgery was 30 months (range: 8–56 months). Biochemical recurrence was observed in 22% (17/77) of patients during the follow-up period. The 3-year biochemical recurrence-free survival rates were 86% (95% CI: 73–93%) for patients with low CETRUS scores and 59% (95% CI: 53–67%) for patients with high CETRUS scores (Fig. 3). Univariate Cox regression analysis revealed that CETRUS score, clinical stage, Gleason score, PSA level, and ECOG PS were significantly correlated with biochemical recurrence-free survival (*p* = 0.015, 0.042, 0.011, 0.037 and 0.047, respectively, Table 3), while other clinicopathologic variables, including age and BMI, were not (*p* = 0.618, 0.205, respectively, Table 3). Using multivariate analysis to further examine the parameters identified as significant by univariate analysis, we determined that patient CETRUS score at the time of diagnosis was an independent predictor of biochemical recurrence (HR: 7.02; 95% CI: 2.00–24.69; *p* = 0.002; Table 3).

**Table 3:** Univariate and multivariate Cox regression analysis of the contribution of potential prognostic factors to biochemical recurrence-free survival of the 77 patients with localized prostate carcinoma who underwent radical prostatectomy

Characteristics	Univariate Analysis		Multivariate Analysis	
	HR (95% CI)	*p*	HR (95% CI)	*p*
Age (≤65 yrs. vs. > 65 yrs)	1.28 (0.49–3.31)	0.618	–	–
CETRUS score (≤3 vs. > 3)	4.11 (1.32–12.74)	**0.015**	7.02 (2.00–24.69)	**0.002**
Tumor stage (T1 vs. T2)	4.66 (1.06–20.53)	**0.042**	5.30 (1.17–24.08)	**0.031**
Gleason score (≤7 vs. > 7)	3.64 (1.35–9.84)	**0.011**	6.38 (2.16–18.88)	**0.001**
PSA (≤15 ng/mL vs. > 15 ng/mL)	2.81 (1.06–7.43)	**0.037**	2.63 (0.74–9.32)	0.135
BMI (< 25 kg/m^2^ vs. ≥25 kg/m^2^)	1.87 (0.71–4.93)	0.205	–	–
ECOG PS (0 vs. ≥1)	2.67 (1.01–7.06)	**0.047**	1.637 (0.46–5.78)	0.444

**Fig. 4:**
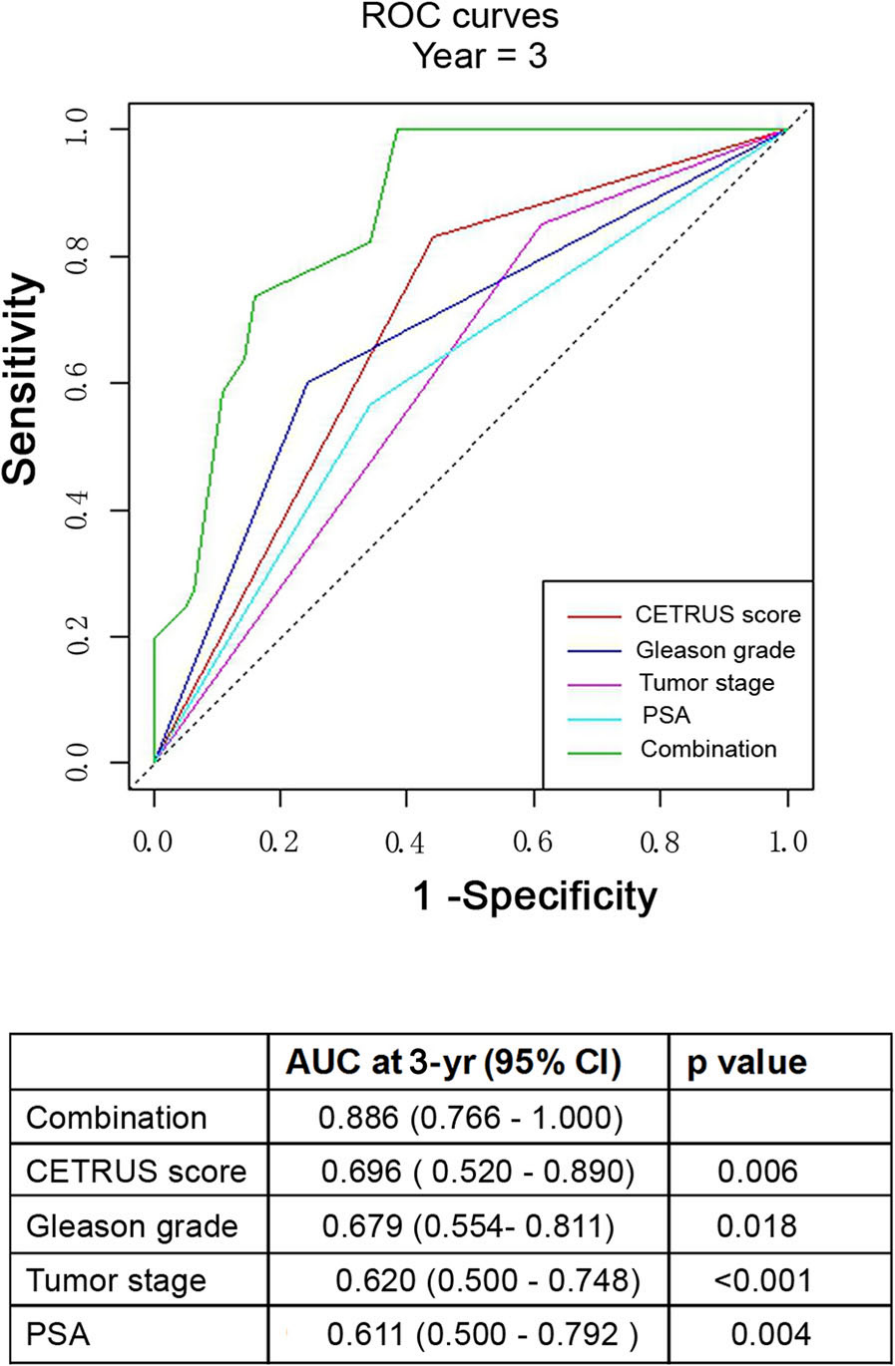
Comparison of the predictive accuracy of different models. *P* values showed the AUC for combined CETRUS score, Gleason grade, tumor stage, and PSA testing at the 3-year mark versus the AUC for CETRUS score alone at the 3-year mark, Gleason score alone, tumor stage alone, or PSA testing alone

**Fig. 5:**
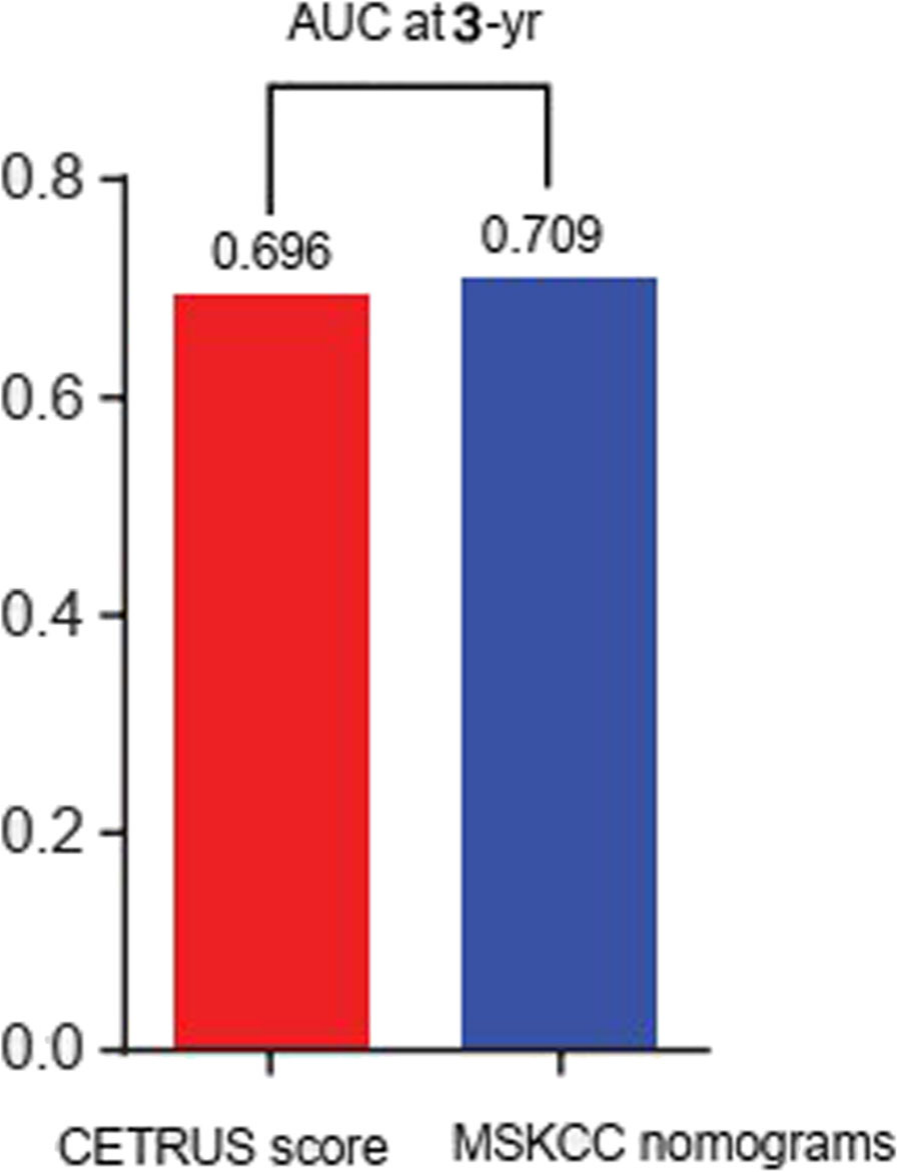
Comparison the 3-year AUC of pre-radical prostatectomy CETRUS scores (0.696) sand MSKCC nomogram (0.709) showed there was no significant difference

To develop a more accurate prognostic tool, we used Cox proportional hazards regression to construct a prognostic model combining CETRUS scores with other clinicopathologic risk factors. A time-dependent ROC curve was used to compare the predictive accuracy of the combined model with the predictive accuracy of CETRUS scores alone or individual clinicopathologic factors alone. As shown in Fig. 4, the model combining patient CETRUS scores, Gleason scores, tumor stage, and PSA levels (AUC at 3 years: 0.886; 95% CI: 0.754–1.000) had a better prognostic value than relying on CETRUS scores alone (AUC at 3 years: 0.696; 95% CI: 0.520–0.890; *p =* 0.006), Gleason scores alone (AUC at 3 years: 0.679; 95% CI: 0.554–0.811; *p =* 0.018), tumor stage alone (AUC at 3 years: 0.620; 95% CI: 0.500–0.748; *p* < 0.001), or PSA levels alone (AUC at 3 years: 0.611; 95% CI: 0.500–0.792; *p =* 0.004). The Memorial Sloan Kettering Cancer Center (MSKCC) nomogram is currently the most commonly used predictor of biochemical recurrence and survival for prostate cancer. Therefore, we compared pre-radical prostatectomy CETRUS scores (0.696) and MSKCC nomograms (0.709) for 3-year AUC and found there was no significant difference between the two methods (Fig. 5). Therefore, CETRUS scores may add prognostic value to the clinicopathologic risk factors currently used in the context of localized prostate cancer.

## Discussion

Our study shows that using CETRUS as a supplement to PSA-based diagnosis can reduce the performance of unnecessary prostate biopsies. We also found that CETRUS scores can independently predict biochemical recurrence of localized prostate cancer following a radical prostatectomy.

PSA testing is not limited to diagnosing prostate cancer. Other conditions, such as benign prostatic hyperplasia and prostatitis, also present with increased PSA levels. Therefore, it is necessary to design screening programs that maximize benefits and minimize morbidity and costs [14]. Angiogenesis, or the process of new blood vessel formation, is necessary for cancer progression. When compared with healthy tissue, the site of prostate cancer is generally characterized by an increased number of newly created blood vessels [15, 16]. However, most of these vessels have small diameters and may not have sufficient flow to be detected by a conventional Doppler transrectal ultrasound. The development of ultrasound contrast agents and contrast-specific imaging techniques have facilitated introduction of CETRUS, a novel imaging modality for continuous visualization of neovascularity associated with prostate cancer [17]. Prostate cancer tissue is often characterized by increased microvessel density due to the proliferation of neovessels. In addition, the microvascular blood supply to prostate tissue is more uniform in malignant tissue than in benign tissue [18].

Multiparametric magnetic resonance imaging (mpMRI) is still the best method for diagnosing prostate cancer. The sensitivity of an MRI guided direct biopsy is as high as 89% of prostate cancer in clinical significance [19]. However, especially in developing countries, this method cannot be widely utilized in many clinics, so the relatively portable B-ultrasound has become another popular tool for diagnosis. Microbubble ultrasound contrast agents allow for the detection of microvessels below the resolution of a conventional transrectal ultrasound. Sedelaar et al. concluded in 2001 that CETRUS has the potential to reveal malignant prostate lesions with increased microvessel density, after finding that enhanced areas on CETRUS had a microvessel density 1.93 times higher than non-enhanced areas [20]. Ultrasound is a real-time, easily accessible, cost-effective, and non-invasive imaging modality. In addition, because ultrasound contrast agents have a low incidence of side effects and are not nephrotoxic, CETRUS can be used in patients with iodine allergies, impaired renal function, or other contraindications that may make them unsuitable for contrast-enhanced computed tomography or MRI [21]. Several studies have shown that the CETRUS-based blood flow grades significantly correlate with the histopathological outcome of biopsy samples. Thus, CETRUS can improve prostate cancer diagnosis, and performance of prostate biopsies based on CETRUS scores represents an innovative approach to detecting significant disease with fewer biopsy cores [8, 9, 22, 23]. With the development of new ultrasound contrast agents and equipment, the diagnostic accuracy of CETRUS is increasing, which will be helpful in eliminating unnecessary biopsy collection in patients without cancer [24]. However, at present, there is limited knowledge of the impact of CETRUS in reducing unnecessary biopsy collection in patients without prostate cancer. In the present study, a second generation contrast agent and low mechanical index were used to improve the survival of microbubbles in the circulation and facilitate real-time depiction of both macrocirculation and microcirculation. In addition, a low mechanical index was used to prolong the time of parenchymal enhancement [25]. The present study showed a strong correlation between CETRUS blood flow grade scores and histopathological findings. Asymmetrical, substantially increased enhancement of the prostate observed during CETRUS was more likely indicate the presence of malignant growth. On the contrary, minimal enhancement with only capsular and periurethral flow was more likely to be benign growth. We found that combining CETRUS scores with additional information besides PSA levels can help predict the result of biopsies in men with elevated PSA. Deciding to perform biopsies on the basis of a CETRUS score ≥ 2 could have reduced the number of biopsies by 12% without missing cancer and spared 23% of men from undergoing an unnecessary biopsy.

Traditional clinicopathologic risk factors are inadequate for accurately predicting the prognosis of patients with localized prostate cancer following a radical prostatectomy. Therefore, additional tools for predicting prostate cancer recurrence may help to identify high-risk patients who might benefit from early intervention. Previous studies have shown that angiogenesis correlates with tumor recurrence following a radical prostatectomy [26–28]. Contrast-enhanced ultrasonography, which is useful for assessing microvessel density, has also been reported to have potential value in predicting the outcome of patients with prostate cancer [29, 30]. Our study demonstrated that CETRUS scores may be a reliable predictor of biochemical recurrence following a radical prostatectomy in patients with localized prostate cancer. CETRUS score successfully categorized patients into high-risk and low-risk subgroups with a significant difference in 3-year biochemical recurrence-free survival. Furthermore, the combination of CETRUS score with others clinicopathologic risk factors had a better prognostic value than CETRUS scores alone or any of the clinicopathologic risk factors alone, suggesting that CETRUS scores complement the prognostic ability of traditional clinicopathologic features. Ultimately, patients with the same stage or grade of localized prostate cancer could potentially be stratified into different CETRUS-score-defined risk groups for disease recurrence, and such stratification may lead to more effective personalized management post-diagnosis and initial treatment.

This study has several noteworthy limitations. First, a 12-core systematic biopsy scheme was used as the reference standard, and this approach may have missed significant tumors. Second, 46% of the men with PCa who underwent a radical prostatectomy had a CETRUS score of ≤3. These patients can easily be mistaken for benign or indeterminate diagnoses. Third, since our study was conducted across only three institutions, the results may not be universally generalizable. We acknowledge that prospective, large-scale, multicenter studies are necessary to confirm our results. With further clinical research confirming the reliability of our research results and improvements to the accuracy of B-ultrasound equipment, performance of B-ultrasounds can not only reduce the puncture rate of patients with localized prostate cancer following a radical surgery, but also reduce unnecessary prostate puncture and biopsy collection by improving the accuracy of prostate cancer screening.

## Conclusions

In summary, CETRUS-based blood flow grading is a reliable tool for reducing unnecessary collection of prostate biopsy samples for prostate cancer diagnosis. This CETRUS-based approach should also be considered to be a novel independent predictor for biochemical recurrence following radical prostatectomy in patients with localized prostate cancer. Our findings indicate that inclusion of CETRUS scores could in the current staging system could add significant prognostic value.

## Supplementary information

**Additional File 1: Table 1**: Diagnostic efficacy of CETRUS scores in differentiating prostate cancer from benign disease at different cut-off points.

**Additional File 2: Figure 1**: Baseline and CETRUS scores 1–5 are depicted in images a-e, respectively.

## Data Availability

The datasets used and/or analyzed during the current study are available from the corresponding author on reasonable request.

## Abbreviations

CETRUS: contrast-enhanced transrectal ultrasound
PSA: prostate-specific antigen
DRE: digital rectal examination
BMI: body-mass index
ECOG PS: Eastern Cooperative Oncology Group performance status
ROC: Receiver operating characteristic
AUC: area under the curve
BPH: benign prostatic hyperplasia
mpMRI: Multiparametric magnetic resonance imaging
MSKCC: Memorial Sloan Kettering Cancer Center.
