# Supplementary material for: Contrast-enhanced transrectal ultrasound can reduce collection of unnecessary biopsies when diagnosing prostate cancer and is predictive of biochemical recurrence following a radical prostatectomy in patients with localized prostate cancer
Source: BMC Urol. 2020 Jul 16;20:100. doi: 10.1186/s12894-020-00659-6 (PMC7364623; doi:10.1186/s12894-020-00659-6)
Supplement: Supplementary file 1 — Additional File 1: Table 1: Diagnostic efficacy of CETRUS scores in differentiating prostate cancer from benign disease at different cut-off points. [file 12894_2020_659_MOESM1_ESM.doc]

**Table S1** Diagnostic efficacy of CETRUS score in differentiating prostate cancer from benign disease at different cut-off points

| **CETRUS score cut-off** | **Sensitivity** | **Specificity** | **Accuracy** |
| --- | --- | --- | --- |
| 2 (=1 as benign and ≥ 2 as malignant) | 53.8% (164/305) | 100% (42/42) | 59.4% (206/347) |
| 3 (≤ 2 as benign and ≥ 3 as malignant) | 62.5% (158/253) | 93.6% (88/94) | 70.9% (246/347) |
| 4 (≤ 3 as benign and ≥ 4 as malignant) | 90.8% (118/130) | 78.8% (171/217) | 83.3% (289/347) |
| 5 (≤ 4 as benign and = 5 as malignant) | 95.0% (57/60) | 62.7% (180/287) | 68.3% (237/347) |
