# Supplementary figures and images for: Contrast-enhanced transrectal ultrasound can reduce collection of unnecessary biopsies when diagnosing prostate cancer and is predictive of biochemical recurrence following a radical prostatectomy in patients with localized prostate cancer
Source: BMC Urol. 2020 Jul 16;20:100. doi: 10.1186/s12894-020-00659-6 (PMC7364623; doi:10.1186/s12894-020-00659-6)

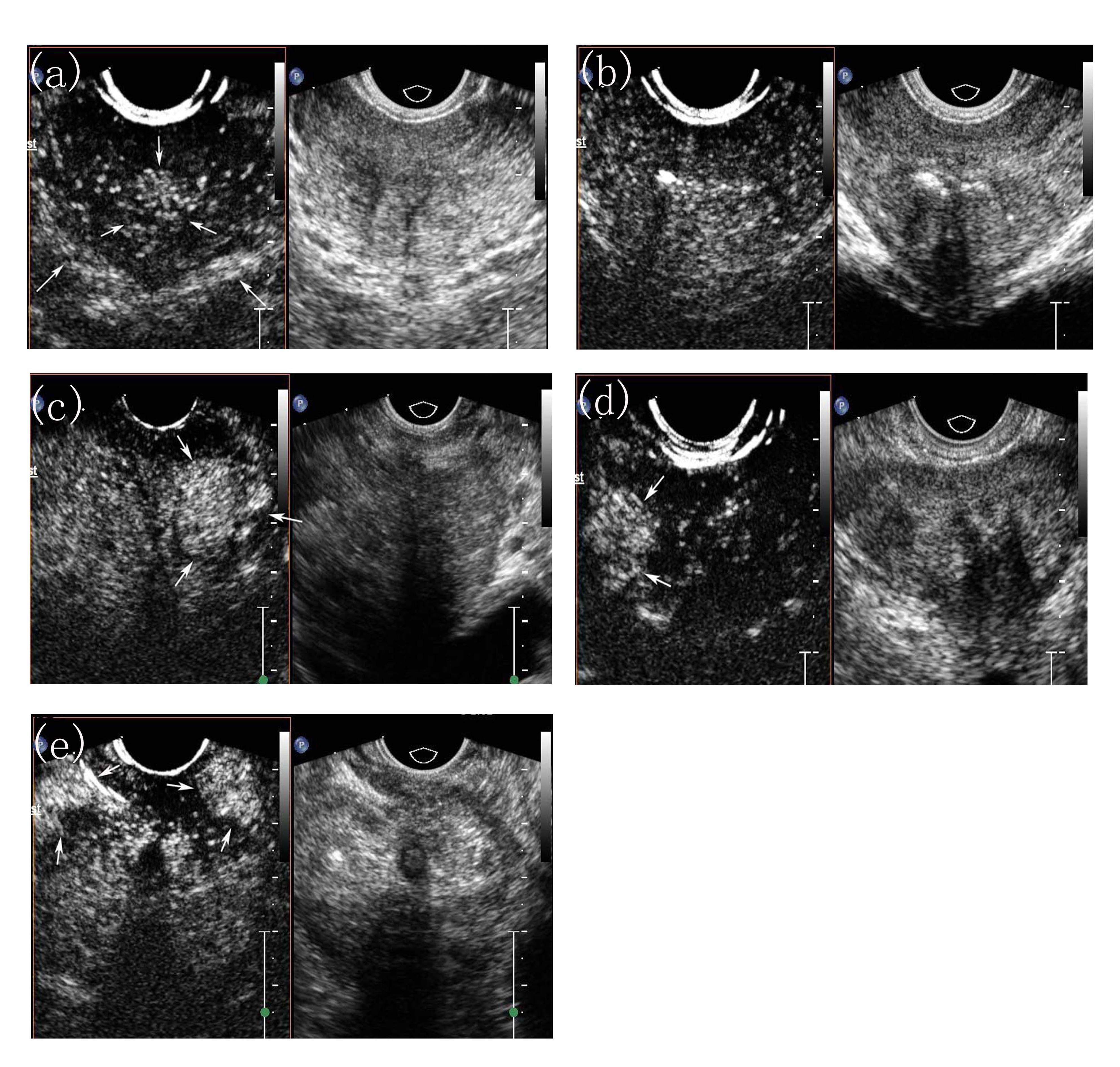

Supplement: Supplementary file 2 — Additional File 2: Figure 1: Baseline and CETRUS scores 1–5 are depicted in images a-e, respectively. [file 12894_2020_659_MOESM2_ESM.jpg]
